# Possible Association of Bovine Gammaherpesvirus 6 with Pulmonary Disease in a Cow

**DOI:** 10.3390/ani13030417

**Published:** 2023-01-26

**Authors:** Selwyn Arlington Headley, Alais Maria Dall Agnol, Thalita Evani Silva Oliveira, Vinícius Rodrigues Bon, Gabriela Sanches Scuisato, Ana Aparecida Correa Xavier, Carolina Yuka Yasumitsu, Alice Fernandes Alfieri, Amauri Alcindo Alfieri

**Affiliations:** 1 Laboratory of Animal Pathology, Department of Veterinary Preventive Medicine, Universidade Estadual de Londrina, Londrina 86057-970, Paraná, Brazil; 2 Multi-User Animal Health Laboratory, Tissue Processing Unit, Department of Veterinary Preventive Medicine, Universidade Estadual de Londrina, Londrina 86057-970, Paraná, Brazil; 3 Programa de Pós-Graduação em Saúde e Produção, Animal, Universidade Pitágoras-Universidade Norte do Paraná, Arapongas 86700-020, Paraná, Brazil; 4 Laboratory of Animal Virology, Department of Preventive Veterinary Medicine, Universidade Estadual de Londrina, Londrina 86057-970, Paraná, Brazil; 5 Multi-User Animal Health Laboratory, Molecular Biology Unit, Department of Veterinary Preventive Medicine, Universidade Estadual de Londrina, Londrina 86057-970, Paraná, Brazil

**Keywords:** bovine respiratory disease, BoGHV6, *Macavirus*, malignant catarrhal fever virus

## Abstract

**Simple Summary:**

Bovine gammaherpesvirus 6 (BoGHV6), previously known as bovine lymphotropic virus, is a member of the *Macavirus* genus, subfamily Gammaherpesvirinae. The role of BoGHV6 in the development of disease in ruminants is controversial. This report identified BoGHV6 in the lungs of a cow with interstitial pneumonia, while other agents associated with the development of pulmonary disease in cattle were not detected by molecular testing. These initial findings suggest that this viral pathogen may be a potential cause of pulmonary disease in cattle.

**Abstract:**

Bovine gammaherpesvirus 6 (BoGHV6), previously known as bovine lymphotropic virus, is a member of the *Macavirus* genus, subfamily Gammaherpesvirinae. Other members of the genus *Macavirus* include viruses that produce malignant catarrhal fever (MCF) in mammalian hosts, collectively referred to as the MCF virus (MCFV) complex, and the porcine lymphotropic herpesvirus (PLHV). However, the current role of BoGHV6 in the development of diseases and/or disease syndromes remains uncertain and controversial. This paper investigated the participation of BoGHV6 in the development of pulmonary disease in a cow with interstitial pneumonia by histopathology and molecular testing. Tissue antigens of common viral agents of respiratory diseases and *Mycoplasma bovis* were not identified by immunohistochemistry. Additionally, molecular assays designed to amplify common bacterial and viral pathogens of pulmonary disease did not amplify the nucleic acids of these agents. However, a pan-PCR assay amplified the DNA of the herpesvirus polymerase gene, while the specific BoGHV6 nested-PCR assay amplified the partial fragment of the BoGHV6 polymerase gene derived from the pulmonary tissue with interstitial pneumonia. Phylogenetic analysis revealed that the BoGHV6 strain herein identified had 99.8% nucleotide (nt) sequence identity with reference strains of BoGHV6, but only 72.2–73.5% and 67.9–68.6% nt identity with reference strains of MCFV and PLHV, respectively. Consequently, these results suggest that BoGHV6 was associated with the pulmonary disease observed in this cow.

## 1. Introduction

Bovine gammaherpesvirus 6 (BoGHV6), formerly referred to as bovine lymphotropic virus, initially isolated from cows with lymphoma [1], is a member of the *Macavirus* genus, subfamily *Gammaherpesvirinae*, family *Herpesviridae* [2]. The *Macavirus* genus contains several members known to produce malignant catarrhal fever (MCF) in their respective mammalian hosts and are collectively referred to as the MCF virus (MCFV) complex since they contain the 15A epitope that is present in all MCFV [3,4], but not in BoGHV6 [5], and probably not in other members of the *Macavirus* genus. Additional members of the *Macavirus* genus include the porcine lymphotropic herpesviruses (PLHVs) [2]. Important MCFV members within the *Macavirus* genus [2] include alcelaphine gammaherpesvirus 1 (AlGHV1) and ovine gammaherpesvirus 2 (OvGHV2), known to produce wildebeest-associated MCF (WA-MCF) and sheep-associated malignant catarrhal fever (SA-MCF), respectively [6,7], and caprine gammaherpesvirus 2 (CpGHV2) associated with MCF in the sika deer [8,9]. While disease processes associated with MCFV are well-established and recognized worldwide, disease syndromes related to infections by BoGHV6 [10] and PLHV [11] are controversial.

The bovine respiratory disease (BRD) complex is a disease entity that is caused by several bacterial and viral pathogens in addition to abrupt management changes and environmental conditions. Bacterial pathogens frequently associated with BRD include *Mannheimia haemolytica*, *Pasteurella multocida*, *Histophilus somni*, and *Mycoplasma bovis* [12,13,14]. The most common viral agents of BRD are bovine viral diarrhea virus (BVDV), bovine respiratory syncytial virus (BRSV), bovine alphaherpesvirus 1 (BoAHV1), bovine parainfluenza virus-3 (BPIV-3), and bovine coronavirus (BoCV) [13,14]. All of these agents have been identified in cattle with BRD from Brazil [14]. Recent studies have shown that a MCFV, or, more likely, OvGHV2 may participate in the development of pulmonary diseases [15,16], by acting individually or in association with other agents of BRD [15]. The possible participation of a MCFV or OvGHV2 in the pathogenesis of BRD was proposed [17] and demonstrated due to the identification of intralesional antigens of a MCFV [15,16] or OvGHV2 [18,19], respectively, in cattle by identifying the monoclonal antibody-15A (MAb-15A) in immunohistochemical (IHC) assays [18].

Since the initial association of BoGHV6 with lymphoma [1], and the proposed participation in the development of bovine leukemia in cows from the USA [20], BoGHV6 was identified in several countries including Belgium [21], Brazil [22], Canada [23], New Zealand [24], Poland [25], and the UK [26,27]. However, the participation of BoGHV6 in the etiopathogenesis of a specific disease entity remains controversial. No association with any disease was described in cows from the USA during a 10-year survey [28], and in cattle from several European countries [29]. Alternatively, BoGHV6 was associated with the development of lymphoproliferative diseases [20,22], endometritis [26,27], metritis [21,24], myocarditis [10], and abortions [23]. Nevertheless, the possible participation of BoGHV6 with the association of pulmonary disease was never investigated. Consequently, this report describes the identification of BoGHV6 in a cow with interstitial pneumonia, providing a possible association between this pathogen and pulmonary disease.

## 2. Detailed Case Description

### 2.1. Tissue Collection and Study Location

Pulmonary samples from an adult beef cattle feedlot cow collected from a slaughterhouse located in the city of Arapongas, approximately 40 km distant from Londrina, Paraná, southern Brazil, were submitted for histopathologic evaluation. This pulmonary sample is part of a larger study *(n* = 180) to identify the possible relationships between histopathologic patterns and the associated infectious disease agents of cattle from Brazil with BRD [15,30]. The pulmonary fragments collected were used for histopathologic analysis using the hematoxylin and eosin (H&E) staining technique. Additionally, selected formalin-fixed paraffin-embedded (FFPE) sections of the lungs were used for the immunohistochemical (IHC) identification of intralesional antigens of agents associated with the development of BRD. Duplicate sections of the lungs were maintained at −80 °C until used in molecular assays.

### 2.2. Immunohistochemical Identification of Pathogens Associated with the Development of BRD 

The FFPE tissue sections of the lungs were used for the immunodetection of intralesional antigens of BRSV, BoAHV1, BVDV, and *M. bovis* [30]. Positive controls consisted of FFPE tissue sections obtained from a previous study [30]. Two negative controls were used: in the first, the primary antibodies were replaced by their corresponding diluents; in the second, FFPE tissues with known negative immunoreactivity to the primary antibodies were immersed with the primary antibodies.

### 2.3. Molecular Detection of Infectious Disease Pathogens of BRD

Nucleic acid extraction from the lung fragment was performed as described using a combination of the phenol/chloroform/isoamyl alcohol and silica/guanidine isothiocyanate methods [31,32]. PCR and RT-PCR assays were carried out to detect the nucleic acids of the of the most frequently occurring infectious disease agents known to cause BRD. These included OvGHV2 [33], BVDV [34], BRSV [35], BoAHV1 [36], BCoV [37], BPIV-3 [38], *M. haemolytica* [39], *P. multocida* [40], *H. somni* [41], and *M. bovis* [42]. For positive controls, the nucleic acids of these disease pathogens agents obtained from previous studies were used: OvGHV2 [18], BVDV, BRSV, BCoV, BRSV, *M. haemolytica*, *P. multocida*, *H. somni*, and *M. bovis* [16]. Additionally, a pan-herpesviruses PCR assay was carried out to amplify the DNA polymerase gene from the pulmonary fragment [43], after which the derived amplicon was submitted to a nested-PCR (nPCR) assay designed to amplify the BoGHV6 polymerase gene [22]. Sterile, ultrapure water was used as the negative control during the extraction of RNA and DNA and in all molecular assays.

### 2.4. Sequencing and Phylogenetic Analysis of BoGHV6 Polymerase Gene

The products of the BoGHV6 polymerase gene amplified from the lung and derived from the specific BoGHV6 PCR assay were purified using a commercial kit (PureLink^®^ Quick Gel Extraction and PCR Purification Combo Kit; Invitrogen^®^ Life Technologies, Carlsbad, CA, USA) and then quantified with the Qubit^®^ Fluorometer (Invitrogen^®^ Life Technologies, Eugene, OR, USA). Thereafter, these products were sequenced with the forward and reverse primers used in each molecular assay in an ABI3500 Genetic Analyzer sequencer with the BigDye Terminator v3.1 Cycle Sequencing Kit (Applied Biosystems^®^, Foster City, CA, USA).

Sequence quality analyses and consensus sequences were obtained using PHRED and CAP3 software (http://asparagin.cenargen.embrapa.br/phph/;accessed 29 October 2021), respectively. Strains of the BoGHV6 polymerase obtained from GenBank were compared with the strain identified during this study using the basic local alignment search tool software (https://blast.ncbi.nlm.nih.gov/Blast.cgi; accessed 29 October 2021). Sequence alignment and identity matrix of the obtained sequences were determined with the BioEdit software [44]. The phylogenetic evaluation was carried out by using the maximum likelihood method with the Kimura 2-parameter model [45] with the MEGA software [46]. The nucleotide sequences of the strain of BoGHV6 identified in this study were compared with the prototype strains for BoGHV6, OvGHV2, AlGHV1 and -2, as well as the nucleotide sequences for CpGHV2 and PLHV.

### 2.5. Clinical, Gross, Histopathologic, and Immunohistochemical Findings

**Figure 1:**
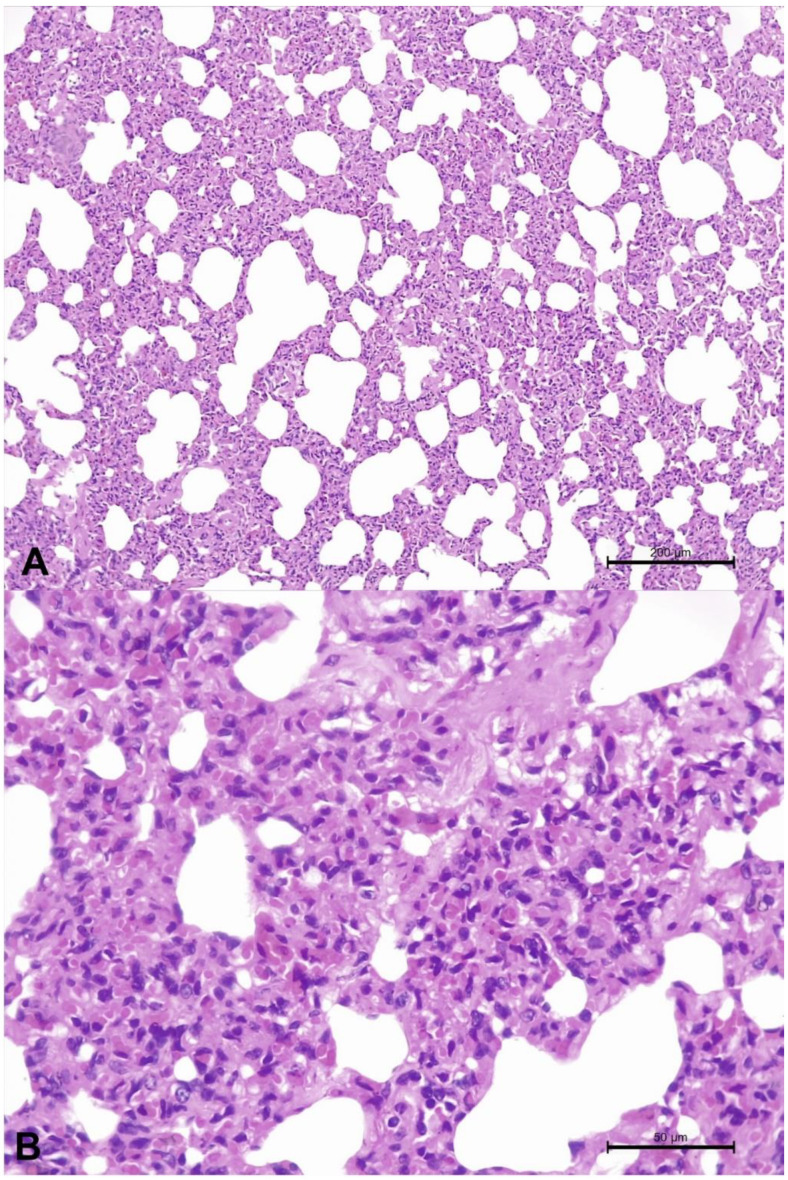
Histopathologic findings observed in a cow infected by bovine gammaherpesvirus 6. There is interstitial pneumonia (**A**) and a closer view demonstrates the thickening of alveolar walls without accumulation of exudate within the alveoli (**B**). Hematoxylin and eosin stain. Bars, (**A**) 200 µm; (**B**) 50 µm.

The clinical data of this animal were unknown since pulmonary tissues were collected at a slaughterhouse. Gross alterations were not observed. Histopathology revealed severe widespread interstitial pneumonia [47] intermingled with patches of normal-looking pulmonary parenchyma. The severe pulmonary disease was characterized by the thickening of alveolar walls due to proliferation of type II pneumocytes admixed with marked accumulations of lymphocytic infiltration (Figure 1A,B). Positive immunoreactivity was not observed for BRSV, BVDV, BoAHV1, and *M. bovis* by IHC.

### 2.6. Molecular Amplification, Sequence, and Phylogenetic Analysis of BoGHV6 Polymerase Gene

**Figure 2:**
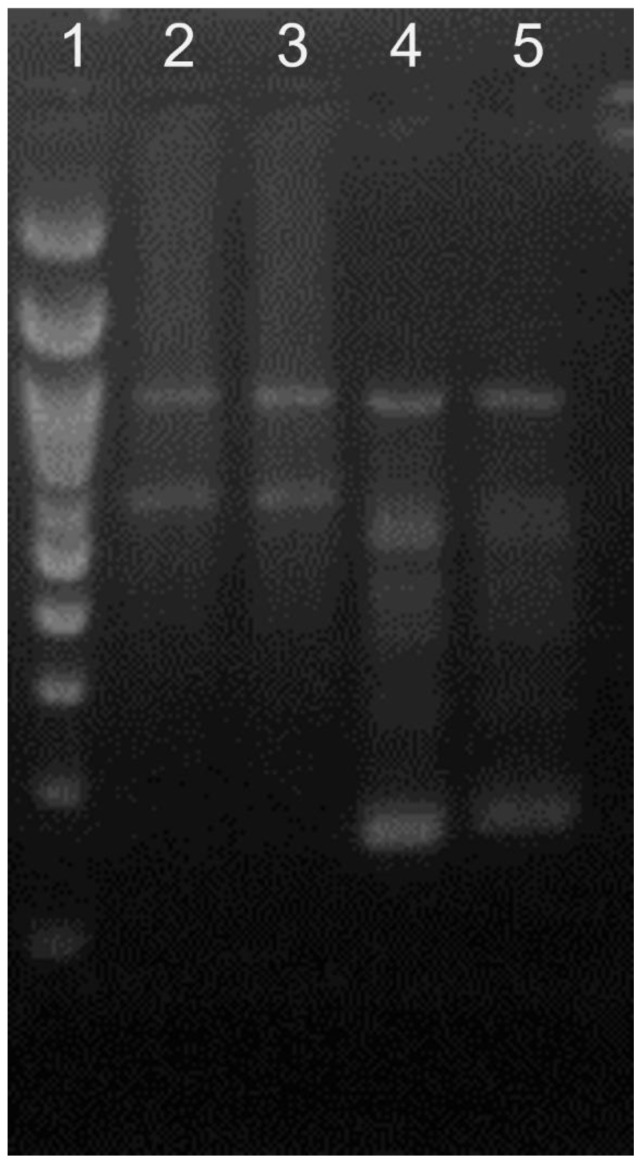
Gel image for the BoGHV6 PCR assays. Lane 1, 100 bp DNA ladder. Lanes 2 and 3, samples in duplicate PCR for BoGHV6 (551 bp); Lanes 4 and 5, samples in duplicate, Nested-PCR for BoGHV6 (166 bp).

The pan-herpes PCR assay, carried out to identify the nucleic acids of any herpesvirus, amplified the DNA of the herpesvirus polymerase gene. Furthermore, the specific BoGHV6 PCR assay amplified the 551 base pairs (bp) fragment of the BoGHV6 polymerase gene derived from the pulmonary tissue with interstitial pneumonia (Figure 2). Additionally, the nt sequence derived from the lung was named BoGHV6/BRA-UEL/PR-538/2019 and deposited in GenBank (GenBank accession # OL310495). Moreover, the molecular assays did not amplify the DNA or RNA of any of the infectious disease pathogens investigated (*M. haemolytica*, *P. multocida*, *H. somni*, *M. bovis*, BRSV, BVDV, BoAHV1, BCoV, BPIV-3, and OvGHV2) that were previously associated with BRD.

**Figure 3:**
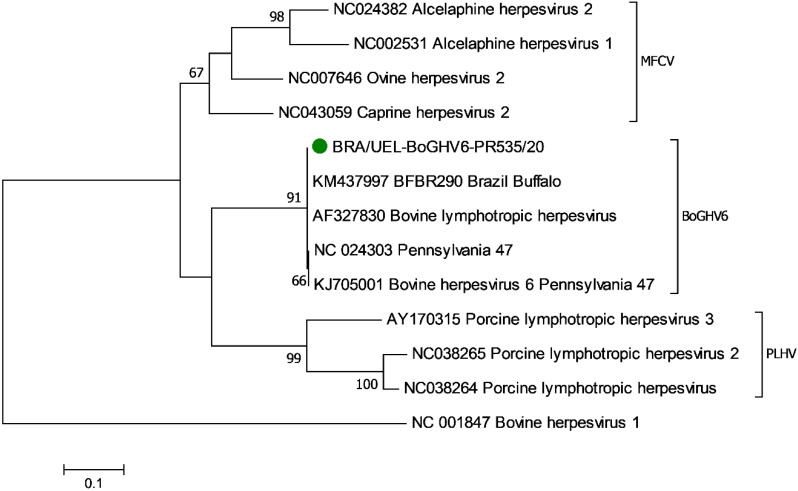
Phylogenetic analysis by the maximum likelihood method of partial DNA polymerase gene of members of the *Macavirus* genus. The evolutionary history was inferred by using the maximum likelihood method based on the Kimura 2-parameter model. The percentage of trees in which the associated taxa clustered together is shown next to the branches. The analysis involved strains of bovine herpesvirus 6 and representative members of each species of the *Macavirus* genus; bovine alphaherpesvirus 1 was used as the out-group. The BoGHV6 strain identified in this study is highlighted (●).

Phylogenetic analyses revealed three definite clusters formed by members (MCFV, BoGHV6, and the PLHV) of the *Macavirus* genus (Figure 3). Furthermore, the BoGHV6 strain identified in this study clustered with members of the BoGHV6 group, being distant from sequences of the MCFV and PLHV. Furthermore, the BoGHV6 strain herein identified had 99.8–100% nt sequence identity with the reference strains (AF327830; NC024303) for BoGHV6 and 100% identity with a strain of BoGHV6 (KM437997) identified in buffalos from Brazil [22]. Additionally, the BoGHV6 strain from this study demonstrated only 72.2–73.5% and 67.9–68.6% nt sequence identity with members of the MCFV and PLHV, respectively.

## 3. Discussion

This study amplified BoGHV6 DNA from the pulmonary fragments of a cow with histopathologic evidence of severe pulmonary disease. Additionally, the nucleic acids of other common bacterial and viral disease pathogens associated with the development of BRD were not amplified, suggesting that these were not associated with the development of the interstitial pneumonia observed by histopathology at the time of tissue collection. Collectively, these findings suggest the possible participation of BoGHV6 in the development of pulmonary disease in this cow and provide a plausible relationship between BoGHV6 and pneumonia. Notwithstanding the above, additional experimental investigations, including in situ diagnostic assays and transinfection studies, must be carried out to investigate the possible role of BoGHV6 in the development of pulmonary disease or any disease syndrome in ruminants, since there is still uncertainty relative to the participation of this agent in infectious disease processes. This is primarily because previous reports have suggested that BoGHV6 may be a commensal agent of cattle [48], not associated with any specific disease [28], an inductor of lymphoproliferative diseases [20,22], or a potential agent of reproductive diseases of ruminants [21,23,24,26,27]. However, in this case, additional organs were not available for evaluation, so the possible identification of BoGHV6 in other tissues from this cow with or without an associated lesion could not have been confirmed or further investigated.

Despite the above, the findings from this study suggest a possible association between BoGHV6 and the development of pulmonary disease in this cow. Additionally, confirmation of the possible association of BoGHV6 and pulmonary disease in cattle has emerged from previous studies by our group. An investigation of the disease pathogens associated with an outbreak of acute respiratory disease in dairy cattle, with clinical manifestation of BRD, identified simultaneous infections of BoGHV6 and OvGH2 in two calves, while one calf was only infected by BoGHV6 [19]. Furthermore, another study by our group that investigated the participation of infectious disease pathogens of BRD in fetal lungs of cattle, identified BoGHV6 DNA in the lungs of four fetuses, during which the lungs of two fetuses had interstitial pneumonia, and BoGHV6 was the only infectious disease agent identified in one of these lungs [49]. Moreover, BoGHV6 was incriminated with the development of intestinal and pulmonary disease in buffalos from Midwestern Brazil [50]. These findings suggest a causal relationship between BoGHV6 and the development of pneumonia in cattle and possibly buffalos. Alternatively, we did not identify BoGHV6 DNA from fetal lungs of cattle that were concomitantly infected by *H. somni* [10]. Collectively, these studies suggest that the role of BoGHV6 in the participation of pneumonia needs further investigation but should not be ignored. Although the role of *Macavirus* in the development of pulmonary disease in ruminants is not certain, we have identified a MCFV, more likely OvGHV2, associated with the development of pulmonary diseases acting individually or in association with other pathogens of lung disease in cattle [15,19]. Furthermore, we have identified OvGHV2 associated with an outbreak of respiratory disease in cattle with clinical manifestations of respiratory distress [19]. Additionally, it was demonstrated that the lungs and lymphatic tissues are probably associated with the initial dissemination of BoGHV6 [29]; similarly, the lungs are the initial sites of replication for OvGHV2 [51,52]. Therefore, it is likely that *Macavirus* may have a similar pathogenesis in the development of pulmonary diseases in mammalians.

The phylogenetic analysis demonstrated that the strain identified in this study is a member of the BoGHV6 group of organisms and demonstrated a distant phylogenetic relationship with MVFV and the PLHV. These findings confirmed that the BoGHV6, MCFV, and PLHV members within the genus *Macavirus*, family *Gammaherpesvirus* are different phylogenetically; similar results were described in [5,20,22]. It must be highlighted that while the disease syndromes associated with MCFV are well-established in mammalian hosts [6,17,51], there is still uncertainty relative to the participation of BoGHV6 [29] and PHLV [11] in the development of diseases in domestic animals.

Additionally, it must be emphasized that this report contrasted with most studies that have investigated the possible association of BoGHV6 with the development of disease syndromes in ruminants. This is because most of the previous studies that have detected BoGHV6 in cattle used only molecular detection without a detailed investigation of the possible histological related findings [20,21,23,24,26,27], while one study related the molecular results with pathological diagnoses observed in organ systems [29]. Alternatively, we have identified BoGHV6 using molecular biology with related histological evidence of pulmonary [49] and myocardial [10] alterations in fetal lungs of cattle as well as in buffalos with histological evidence of pulmonary and intestinal alterations [50]. This collectively suggests that most previous studies that detected BoGHV6 in ruminants have not investigated the possibility of this virus being able to produce any histological evidence of disease. Therefore, further studies must be based on the in-situ detection of BoGHV6 in tissues with histologic evidence of disease to determine the role of this virus in the development of disease and/or histological alterations in ruminants.

## 4. Conclusions

These findings demonstrated that BoGHV6 DNA was amplified from the pulmonary tissues of a cow with interstitial pneumonia. Phylogenetic analyses confirmed that the strain identified within the pulmonary tissues has 100% nt identity with reference strains of BoGHV6 but was phylogenetically different from MCFV. These initial findings suggest that this virus may be a potential agent associated with the development of respiratory disease in cattle.

## Data Availability

The nucleotide sequence of the BoGHV6 strain identified during this study is deposited in GenBank (https://www.ncbi.nlm.nih.gov/genbank/, accessed on 4 October 2022).
